# Supplementary material for: Using repeated home-based HIV testing services to reach and diagnose HIV infection among persons who have never tested for HIV, Chókwè health demographic surveillance system, Chókwè district, Mozambique, 2014–2017
Source: PLoS One. 2020 Nov 20;15(11):e0242281. doi: 10.1371/journal.pone.0242281 (PMC7678994; doi:10.1371/journal.pone.0242281)
Supplement: S1 File — (DOCX) [file pone.0242281.s001.docx]

**S1 File.**

**S1: Composite variables**

Knowledge about HIV included the following 9 items:

- A healthy-looking person can have HIV.
- HIV can be transmitted from a mother to her baby during pregnancy.
- HIV can be transmitted from a mother to her baby during delivery.
- HIV can be transmitted from a mother to her baby during breast feeding.
- Condom use can reduce HIV risk.
- Circumcision for an HIV-negative man can reduce the risk of getting HIV.
- Circumcision for an HIV-positive man can reduce the risk of giving HIV.
- Antiretroviral therapy (ART) is available for treating HIV infection.
- ART has benefits for HIV-infected persons and reduces their risk for transmitting HIV to their partner.

Each item received 1 point for a correct response and 0 points for an incorrect or do-not-know response; thus, the maximum score was 9. The Cronbach alpha coefficient for this measure was 0.65.

Beliefs about ART included the following 6 items:

- HIV-infected persons can live a long, normal life if they take ART.
- Traditional medicine is as good as ART.
- Persons taking ARV medicines need to hide the medicines so that other people won’t find out.
- ART is only given to HIV-infected persons who are feeling really bad.
- After testing HIV-positive, immediate HIV care is not needed if the person is feeling good.
- Special kinds of drugs can be given by a doctor or nurse to HIV-infected pregnant women to reduce the risk for HIV transmission to the baby.

Each item received 1 point for a correct response and 0 points for an incorrect or do-not-know response; thus, the maximum score was 6. The Cronbach alpha coefficient for this measure was 0.85.

HIV stigma included the following 8 items:

- Family members of persons living with HIV/AIDS should be ashamed.
- Persons with AIDS should be isolated from other people.
- Persons living with HIV/AIDS deserve to be punished.
- Persons living with HIV/AIDS should be ashamed.
- Persons living with HIV/AIDS in this community face rejection from their peers.
- Persons living with HIV/AIDS in this community face verbal abuse or teasing.
- Persons living with HIV/AIDS in this community face rejection from their home by their family.
- Persons living with HIV/AIDS in this community face neglect from their family.

Each item was scored on a 2-point scale: disagree = 0; neutral = 1; agree = 2; thus, the maximum score was 16. The Cronbach alpha coefficient for this measure was 0.66.
