# Supplementary material for: Using repeated home-based HIV testing services to reach and diagnose HIV infection among persons who have never tested for HIV, Chókwè health demographic surveillance system, Chókwè district, Mozambique, 2014–2017
Source: PLoS One. 2020 Nov 20;15(11):e0242281. doi: 10.1371/journal.pone.0242281 (PMC7678994; doi:10.1371/journal.pone.0242281)
Supplement: S2 File — (DOCX) [file pone.0242281.s002.docx]

**Survey Questions**

| **Portuguese** | **English** | **VAR** |
| --- | --- | --- |
| Hilihi rambu lawena?  (1. Wanuna; 2. Wansati) | Sex of participant?  (1. Male; 2. Female) | SEX |
| Una malembe mangaki yakuvelekiwa? | How old are you in complete years? | AGE |
| Utekile/utekiwile?  (1.Andzisiteka/andzisitekiwa 2. Utxadile; 3.Andzibalisanga awukati; 4.Ndzidlayi ukati; 5.Hitsikanile; 6. Mufelakzi) | What is your current marital status?  (1. Single; 2. Married; 3. Marital Union; 4. Divorced; 5. Separated;  6. Widow/er) | MARITAL |
| I vanhu vangaki uvativaka vanganfa navani SIDA?  (8888 Svihava ka nxanxameto; 9999. Andzi swi tivi) | How many people do you know who have died of AIDS?  (8888. Not applicable; 9999. Don’t know) | KNWLDG3 |
| Ukhanga ufamba masangu?  (1. Ina; 2.Ahim him; 99. Andziswitivi) | Have you ever had sexual intercourse?  (1. Yes; 2. No; 99. Don’t know) | SEX1 |
| Ka khume la tiwheti na timbirhi ta wugamu ufambelani ni vanhu vangaki vahanyaka amigangeni leyi, **(SIZA UXAXAMETA HINKWAVU, NAMBHI LAVA UNGAFAMBELANAA NAVONA HINKARHINYANA ).**  Ivangaki lava ungafambelana navona vatsamaka kumbe vatsamiki ka miganga leyi:  a)Kaxifundza xaka Chókwè  b) Ka xifundza xaka Gaza kumbe handle ka Chókwè?  c)Lomu Moçambique, kambe handle ka xifundza xaka Gaza?  d) A djoni?  E) Linwana tiku  _______________________________?  (88. Swihava ka nxanxameto; 99.Andziswitivi) | In the past 12 months, with how many partners who live in the following areas have you had sexual intercourse? PLEASE INCLUDE ALL PARTNERS INCLUDING CASUAL PARTNERS.  How many of your sexual partners live or lived in:   1. Chókwè District? 2. Gaza Province, but not in Chókwè District)? 3. In Mozambique, but not in Gaza Province? 4. In South Africa? 5. In another country:____________________________?   (8888. Not applicable; 9999. Don’t know) | SEX2  SEX3  SEX4  SEX5  SEX6 |
| Munhu lwe aali nsati wa wena, munhu waku mukuma hi nkarhinyana, kunbe waku xintxana?  (1. Nsati wa wena = kuvuliwa lwe utxadiki nayena, kumbe lwe uhanyaka nayena unga mitxandanfga; 2.Munhu waku mukuma hinkarinyana= ufambelaniki nayena amasagu khambi linwe, kumbe mankhambe yakukalakunyawula;  3.Munhu waku xintxana = Hilwe ungafambelana nayena masangu angali nsati wa wena futhi angali xigangu xankarhinyana, wu yohakela, kumbe yena ahakeli wena akuva mifamaba amasangu  (88. Swihava ka nxanxameto; 99.Andziswitivi) | Is this last person with whom you had sex a spouse, or a casual or exchange partner?  (1. Spouse = married or living with as if married; 2. Casual partner = someone with whom participant had sex only once, a few times, or only occasionally;  3. Exchange partner = partner who is not a steady or casual partner who was paid or who paid participant to have sex; 88. Not applicable; 99. Don’t know) | SEX7 |
| Tsundzuka, khambhi lawugamu ufambelaniki nayena munhu lweyi,utirhisi xithlangu?  ( 1.Ina; 2.Ahim him; 88. Swihava ka nxanxameto; 99.Andziswitivi) | Think back to the very last time you had sex with this partner.  Was a condom used the last time you had sex with him/her?  (1. Yes; 2. No; 88. Not applicable; 99. Don’t know) | SEX10 |
| Ukhangi umuvutisa mayelanu ni HIV?ni lesvaku akhanga ayendla xikambelo xa HIV?  ( 1.Ina; 2.Ahim him; 88. Swihava ka nxanxameto; 99.Andziswitivi) | Did you ever ask about his/her HIV status or whether he/she has tested for HIV?  (1. Yes; 2. No; 88. Not applicable; 99. Don’t know) | SEX11 |
| Ka tiwheti tinharhu leti ta wugamu udzahile timbangi takufana ni leti?   1. Namarijuana (Suruma/nbhangi)   b) Kumbe tinwana  (1.Ina; 2.Ahim him; 88. Swihava ka nxanxameto; 99.Andziswitivi) | In the last three months, have you used any of the following drugs?   1. Marijuana 2. Other   (1. Yes; 2. No 88. Not applicable; 99. Don’t know) | DRUG1  DRUG2 |
| Ka khume la tiwethi natimbiri leti ta wugamu ukhangi uhumela nsila hika xirhu xawena (xaxitasi kumbe xaxinuna) Siza unga phati lesvi svi humaka loko munhu afamba massango  (1.Ina; 2.Ahim him; 88. Swihava ka nxanxameto; 99.Andziswitivi) | In the past 12 months, have you had abnormal or unusual discharge from the vagina (women)/penis (men)? Please do not include discharge during sexual intercourse.  (1. Yes; 2. No; 88. Not applicable; 99. Don’t know) | STI2 |
| Ka khume la tiwheti na timbirhi leti ta wugamu, kuni mun’we ka lava ufambelanaka navona masangu akhangiki akuba, kukunyika mutapi, kumbe ayendla xilo xaku kuvavisa?  (1.Ina; 2.Ahim him; 88. Swihava ka nxanxameto; 99.Andziswitivi) | In the last 12 months, have any of your sexual partners hit, slapped, kicked, or done anything else to hurt you physically?  (1. Yes; 2. No; 88. Not applicable; 99. Don’t know) | VLNCE1 |
| Ka khume la tiwheti leti ta wugamu, kuni lwe akhangiki akusindzisa akufamba amasangu, kumbe svinwana svakuyelana ni masangu?  (1.Ina; 2.Ahim him; 88. Swihava ka nxanxameto; 99.Andziswitivi) | In the last 12 months, have any of your sexual partners forced you in any way to have sexual intercourse or perform any other sexual acts?  (1. Yes; 2. No; 88. Not applicable; 99. Don’t know) | VLNCE2 |
| Swinga endla munhu ani matsamela manene na ali ni xitsongwatsongwana xa SIDA?  (1. Ina; 2. Ahi him; 88. Swihava ka nxanxameto 99. Andzi swi tivi) | Is it possible for a healthy-looking person to have the AIDS virus?  (1. Yes; 2. No; 88. Not applicable; 99. Don’t know) | KNWLDG4 |
| Wansati lwe angani Xitsongwatsongwani lexi xi vangaka SIDA anga tluleta a nwana wa yena:  a) A nyimbeni?  b) Nkari lowu aphulukaka?  c)Nkari lowu amamisaka?  (1. Ina; 2. Ahi him; 88. Swihava ka nxanxameto; 99.Andziswitivi) | Can the virus that causes AIDS be transmitted from a mother to her baby:   1. During pregnancy? 2. During delivery? 3. By breastfeeding?   (1. Yes; 2. No; 88. Not applicable; 99. Don’t know) | KNWLDG5  KNWLDG6  KNWLDG7 |
| Vanhu va nga pumba a mhangu yakukuma xitsongwatsongwani lexi xi vangaka a SIDA loko va tirhisa a xithlangu, kama ni kama va fambaka masangu?  (1. Ina; 2.Ahim him; 88. Swihava ka nxanxameto; 99. Andziswitivi). | Can people reduce their chance of getting the AIDS virus by using a condom every time they have sex?  (1. Yes; 2. No; 88. Not applicable; 99. Don’t know) | KNWLDG9 |
| Awanuna lwe anga hava a xitsongwatsongwani lexi xi **vangaka** a mavabyi loko a susiwa nsuvo a mhangu ya kukuma HIV yokula, yopumbeka, kumbe yonhima kola ka khale?  (1. Yoyengeteleka; 2.Yopumbeka; 3. Yonyima kola ka khale? 99.Andziswitivi) | When a man without HIV becomes circumcised, does his risk for GETTING HIV increase, decrease, or remain about the same?  (1. Increase; 2. Decrease; 3. Remain the same; 99. Don’t know) | KNWLDG10 |
| Loko wanuna lwe angani xitsongwatsongwani (HIV), asusiwa nsuvo a mhangu **yakutluleta** a HIV yokula, yopumbeka, kumbe yonhima kola ka khale?  (1. Yoyengeteleka; 2.Yopumbeka; 3. Yonyima kola ka khale? 99.Andziswitivi) | When a man with HIV becomes circumcised, does his risk for GIVING HIV increase, decrease, or remain about the same?  (1. Increase; 2. Decrease; 3. Remain the same; 99. Don’t know) | KNWLDG11 |
| Ukhanga usviyingela navavulavula himimirhi (amanterotrovirais) leyi vayinyikaka munhu lwe angani xitsongwatsongwani lexi xi vangaka a SIDA anga nyikiwaka hi dokodela kumbe enfermeiro? (1. Ina; 2.Ahim him; 88. Svihava ka nxanxameto; 99.Andziswitivi). | Have you heard about ARV medicines (USE LOCAL NAME) that people infected with the AIDS virus can get from a doctor or a nurse?  (1. Yes; 2. No; 88. Not applicable; 99. Don’t know) | KNWLDG12 |
| Loko munhu lwe angani HIV aphuza a mimirhi (amanterotrovirais) mhangu **yakutluleta** ka munwana yokula, yopumbeka, kumbe yonhima kola ka khale? 1. Yoyengeteleka; 2.Yopumbeka; 3. yonyima kola ka khale? 88. Swihava ka nxanxameto; 99.Andziswitivi) | When a person with HIV takes these ARV medicines (USE LOCAL NAME), does his or her risk of GIVING HIV to a sexual partner increase, decrease, or remain about the same?  (1. Increase; 2. Decrease; 3. Remain the same; 99. Don’t know) | KNWLDG13 |
| Wena **wapfumela** kumbe **awupfumeli** leswi:  a)Vanhu lava vangani a HIV vanga hanya mpfuka wo leha na va hanya khwatsi loko va phuza a mimirhi (amantirovirais)  b)A mimirhi ya xintu na yona yinene akufana ni amantirovirais aku yetleliseni ka xitsongwatsongwani lexi xi vangaka a SIDA.  c) Amantirovirais mafaneli aku phuziwa ntsena hi munhu lwe avabyaka ngopfu.  d)Vanhu lava vaphuzaka amantirovirais vafaneli akumafihla akuva vanhu vanwana vanga switivi.  e)Loko munhu akumiwa leswaku ani xitsongwatsongwani (HIV), aswifanela akuva a sungula akulandza minawu ya timbabyi, loko ahatiyingela kahle.  f)Kuni mimirhi leyi dokodela kumbe enfermeiro anganyikaka wansati lwe angani kwirhi na a ni HIV, akuva yi sivela akuva xitsongwatsongwani lexi xitlulela a xinwanana.  (1. Ina; 2.Ahim him; 88. Swihava ka nxanxameto; 99. Andziswitivi). | Do you agree or disagree with the following statements?   1. Persons with HIV can live a long and healthy life if they take ARV medicines. 2. Traditional medicine is just as good as ARV medicines in treating HIV/AIDS. 3. ARV medicines are only given to persons with HIV who are feeling really bad. 4. Persons taking ARV medicines need to hide the medicines so that other people won’t find out. 5. After testing HIV positive, there is no need for persons to immediately get HIV care if they are feeling good. 6. There are special drugs that a doctor or a nurse can give to a woman infected with the AIDS virus to reduce the risk of transmission to the baby.   Agree; 2. Disagree; 88. Not applicable; 99. Don’t know) | BELIEF1  BELIEF2  BELIEF3  BELIEF4  BELIEF5  BELIEF6 |
| Wena wapfumela kumbe awupfumeli leswi:  Mindjangu ya vanhu lava va hanyaka ni xitsongwatsongwana xa HIV va fanela ku vakhomiwa hi tingana.  Vanhu lava va hanyaka ni xitsongwatsongwana xa HIV va nfanele kulhauliwa vangahanyi ni vanhu vanwana.  Vanhu lava va hanyaka ni xitsongwatsongwana xa HIV vafaneli kukhatisiwa.  Vanhu lava va hanyaka ni xitsongwatsongwana xa HIV vafaneli kukhomiwa hi tingana.  Vanhu lava vahanyaka ni xitsongwatsongwana xa HIV, amugangeni lowu, vakhetiwa hi wanganu afana ni vanghanu (va nwani).  Vanhu lava va hanyaka ni xitsongwatsongwana xa HIV, amugangeni lowu vapoyiliwa vatlela varukiwa.  Amugangeni lowu, vanhu lava vahanyaka ni xitsongwatsongwana (HIV) vahlongoliwi hi maxaka amitini ya vona.  Amugangeni lowu, vanhu lava vahanyaka ni xitsongwatsongwana (HIV) avahlayisiwi hi maxaka ya vona.  1=SD, 2=D, 3=N, 4=A, 5=SA | Do you agree or disagree with the following statements?  Families of people living with HIV/AIDS should be ashamed.  People with AIDS should be isolated from other people.  People living with HIV/AIDS deserve to be punished.  People living with HIV/AIDS should be ashamed.  People living with HIV/AIDS in this community face rejection from their peers.  People who have HIV/AIDS in this community face verbal abuse or teasing.  People living with HIV/AIDS in this community face ejection from their homes by their families.  People living with HIV/AIDS in this community face neglect from their family.  1=SD, 2=D, 3=N, 4=A, 5=SA | STG1  STG2  STG3  STG4  STG5  STG6  STG7  STG8 |
| Ukhangi uyendla xikambelo xa HIV?  (1.Ina; 2.Ahim him; 88. Swihava ka nxanxameto; 99.Andziswitivi) | Have you ever tested for HIV?  (1. Yes; 2. No; 88. Not applicable; 99. Don’t know) | TEST1 |
| Awukhangi uyendla axikambelo xa HIV hayini?   \| **[UNGA HLAYI: VEKA HI MACÓDIGO YA NXANXAMETO]**  a)Aninga lika mhango yakukuma HIV \| \| --- \| \| b)Ndzi txava kutiva lesvaku ndzini HIV \| \| c)Loko ndzini HIV, ndzingaluza lwe ndzifambelanaka nayena masangu, maxaka, kumbe vanghanu \| \| d)Loko ndzini HIV ndzinga biwa kumbe ndzi vavisiwa hi lweyi ndzifambelanaka nayena masangu \| \| e)Lweyi ndzifambelanaka nayena masangu angaswilavi akuva ndziyendla xikambelo \| \| f)Maxaka ni vanghanu awasvilavi akuva ndziyendla xikambelo \| \| g)Ndzihanya kule ni lomu vayendlaka kona swikambelo \| \| h)Swa durha akuyendla xikambelo \| \| i)Vatirheli va udahi avakhangi varhamba vanhu akuva vaendla xikambelo \| \| j)Andziyitivi ndhawu lomu vaendlaka kona svikambelo \| \| k) Swinwana____________________________ \| \| (1.Ina; 2.Ahim him; 88. Swihava ka nxanxameto; 99.Andziswitivi ) \| | Why have you not tested for HIV?  [DO READ RESPONSES; CODE ALL THAT APPLY]   \| 1. Not at risk for HIV \| \| --- \| \| 1. Afraid to learn he/she is HIV-positive \| \| 1. If HIV+, will lose partner/family/friends \| \| 1. If HIV+, will be beaten/hurt by partner \| \| 1. Partner does not want me to test \| \| 1. Family/friends do not want me to test \| \| 1. Live too far from where he/she can be tested \| \| 1. Costs too much money to test \| \| 1. Health provider never offered test \| \| 1. Didn’t know where to test for HIV \| \| 1. Other_________________________________ \| \| (1. Yes; 2. No; 88. Not applicable; 99. Don’t know) \| | TEST2  TEST3  TEST4  TEST5  TEST6  TEST7  TEST8  TEST9  TEST10  TEST11  TEST12 |
| Ka khume la tiwheti natimbirhi leti landzaka uni makungu yakuyendla xikambelo xa HIV?  (1.Ina; 2.Ahim him; 88. Swihava ka nxanxameto; 99.Andziswitivi) | Do you intend to test for HIV in the next 12 months?  (1. Yes; 2. No; 88. Not applicable; 99. Don’t know) | TEST13 |
| Hikwihi lomu uyendliki kona xikambelo xa HIV, khambi lawugamu?  (1. Xibelheli ka Chókwé; 2. Amutini wa mina ka Chókwé ; 3. Antirwheni wa mina ka Chókwé ; 4 Tinwana tindhawu ka Chókwé ; 5 Tinwana tindhawu ka Gaza, kambe handle ka Chókwé; 6.Yinwana ndhawu Moçambique, kambe handle ka Gaza; 7. Tiku linwana; 88. Svihava ka nxanxameto; 99.Andzisvitivi) | Where did you test the very last time you tested for HIV?  (1. Hospital or clinic in Chókwè District; 2. Home in Chókwè District ; 3. Work in Chókwè District ; 4 Other location in Chókwè District ; 5. Other location in Gaza Province, but not in Chókwè District; 6. Other location in Mozambique, but not in Gaza Province; 7. Other country; 88. Not applicable;  99. Don’t know) | TEST18 |
