# Supplementary material for: Using repeated home-based HIV testing services to reach and diagnose HIV infection among persons who have never tested for HIV, Chókwè health demographic surveillance system, Chókwè district, Mozambique, 2014–2017
Source: PLoS One. 2020 Nov 20;15(11):e0242281. doi: 10.1371/journal.pone.0242281 (PMC7678994; doi:10.1371/journal.pone.0242281)
Supplement: S1 Table — (DOCX) [file pone.0242281.s003.docx]

**S1 Table: Demographic and behavioral factors associated with never tested for HIV before by weighted approach**

|  | 2014 |  | 2015 |  | 2016 |  | 2017 |  |
| --- | --- | --- | --- | --- | --- | --- | --- | --- |
|  | **Crude OR**  **(95% CI)** | **Adjusted OR (95% CI)** | **Crude OR**  **(95% CI)** | **Adjusted OR (95% CI)** | **Crude OR**  **(95% CI)** | **Adjusted OR**  **(95% CI)** | **Crude OR**  **(95% CI)** | **Adjusted OR (95% CI)** |
| Sex |  |  |  |  |  |  |  |  |
| Male | 2.58 (2.15, 3.10) |  | 2.12 (1.66, 2.73) |  | 2.16 (1.69, 2.76) |  | 1.52 (1.15, 2.02) |  |
| Female | 1 |  | 1 |  | 1 |  | 1 |  |
| Age |  |  |  |  |  |  |  |  |
| <18 | 4.41 (3.49, 5.60) |  | 4.93 (3.64, 6.67) |  | 7.51 (5.55, 10.16) |  | 16.47 (10.63, 25.54) | 6.56 (3.77, 11.41) |
| 18-24 | 1.50 (1.21, 1.85) |  | 1.20 (0.84, 1.71) |  | 1.19 (0.80, 1.76) |  | 0.84 (0.46, 1.55) | 0.61 (0.31, 1.20) |
| ≥ 25 | 1 |  | 1 |  | 1 |  | 1 | 1 |
| Relationship |  |  |  |  |  |  |  |  |
| Other | 2.16 (1.81, 2.60) |  | 2.70 (2.04, 3.57) |  | 2.57 (1.94, 3.41) |  | 5.78 (3.81, 8.79) |  |
| Married/martial union | 1 |  | 1 |  | 1 |  | 1 |  |
| Knowing people died with AIDS |  |  |  |  |  |  |  |  |
| No | 1.69 (1.38, 2.08) |  | 1.61 (1.10, 2.32) |  | 1.22 (0.82, 1.81) |  | 2.02 (1.16, 3.51) |  |
| Yes | 1 |  | 1 |  | 1 |  | 1 |  |
| Ever have sex |  |  |  |  |  |  |  |  |
| No | 3.93 (3.01, 5.12) | 6.28 (3.98 , 9.90) | 5.11 (3.81, 6.87) | 5.85 (3.56, 9.63) | 5.41 (4.14, 7.04) |  | 11.68 (8.47, 16.11) | 4.60 (2.54, 8.34) |
| Yes | 1 | 1 | 1 | 1 | 1 |  | 1 | 1 |
| Having casual sex or exchange partner (last person had sex with) |  |  |  |  |  |  |  |  |
| No | 0.68 (0.55, 0.83) | 0.69 (0.54, 0.90) | 0.76 (0.56, 1.03) | 0.60 (0.39, 0.92) | 1.16 (0.86, 1.56 ) |  | 1.50 (1.05, 2.14) |  |
| Yes | 1 | 1 | 1 | 1 | 1 |  | 1 |  |
| Having unprotected sex (no condom) with last sex partner |  |  |  |  |  |  |  |  |
| No | 1.27 (1.04, 1.55) | 0.52 (0.39,0.69) | 2.08 (1.60, 2.72) |  | 2.26 (1.74, 2.93) |  | 4.44 (2.98, 6.60) |  |
| Yes | 1 |  | 1 |  | 1 |  | 1 |  |
| Never asked partner about HIV status when have sex |  |  |  |  |  |  |  |  |
| No | 0.47 (0.39, 0.56) | 0.32 (0.25, 0.41) | 0.79 (0.61, 1.04) | 0.35 (0.23, 0.53) | 0.83 (0.64, 1.09) | 0.38 (0.24, 0.61) | 1.42 (0.99, 2.01) | 0.34 (0.19, 0.62) |
| Yes | 1 | 1 | 1 | 1 | 1 | 1 | 1 | 1 |
| Having STI in the past 12 month |  |  |  |  |  |  |  |  |
| No | 1.84 (1.48, 2.29) |  | 1.92 (1.29, 2.85) |  | 1.55 (1.00, 2.39) |  | 5.40 (2.21, 13.17) |  |
| Yes | 1 |  |  |  |  |  | 1 |  |
| Drug use |  |  |  |  |  |  |  |  |
| No | 0.53 (0.36, 0.78) |  | 0.53 (0.25, 1.11) | 0.47 (0.20, 1.11) | 0.46 (0.23, 0.91) |  | 0.66 (0.24, 1.78) |  |
| Yes | 1 |  | 1 | 1 | 1 |  | 1 |  |
| Score: Knowledge about HIV | 0.84 (0.80, 0.89) | 0.88 (0.83, 0.94) | 0.81 (0.75, 0.87) | 0.89 (0.82, 0.97) | 0.76 (0.72, 0.81) | 0.87 (0.81, 0.93) | 0.75 (0.69, 0.81) | 0.90 (0.82, 0.99) |
| Score: believe of ARV | 0.88 (0. 84, 0.92) |  | 0.87 (0.82, 0.93) |  | 0.87 (0.81, 0.93) |  | 0.83 (0.76, 0.90) |  |
| Score: stigma | 1.02 (0.98, 1.08) |  | 1.13 (1.05, 1.21) |  | 1.11 (1.06,1.18) | 1.10 (1.03, 1.18) | 1.01 (0.90, 1.14) |  |
| Interaction: age and gender |  |  |  |  |  |  |  |  |
| Age<18 and male |  | 3.54 (2.32, 5.40) |  | 2.42 (1.42, 4.12) |  | 13.67 (5.46, 34.26) |  |  |
| Age<18 and female |  | 5.15 (3.30, 8.04) |  | 5.41 (3.46, 8.48) |  | 13.06 (5.26, 32.38) |  |  |
| Age>=18 and male |  | 3.48 (2.70, 4.50) |  | 3.12 (2.07, 4.72) |  | 3.31 (2.07, 5.31) |  |  |
| Age>=18 and female |  | 1 |  | 1 |  | 1 |  |  |
| Interaction: age and ever have sex |  |  |  |  |  |  |  |  |
| Age<18 and never have sex |  |  |  |  |  | 12.00 (7.56, 19.05) |  |  |
| Age<18 and have sex |  |  |  |  |  | 3.26 (2.00, 5.32) |  |  |
| Age>=18 and never have sex |  |  |  |  |  | 0.73 (0.16, 3.28) |  |  |
| Age>=18 and have sex |  |  |  |  |  | 1 |  |  |
