# Supplementary material for: Using repeated home-based HIV testing services to reach and diagnose HIV infection among persons who have never tested for HIV, Chókwè health demographic surveillance system, Chókwè district, Mozambique, 2014–2017
Source: PLoS One. 2020 Nov 20;15(11):e0242281. doi: 10.1371/journal.pone.0242281 (PMC7678994; doi:10.1371/journal.pone.0242281)
Supplement: S2 Table — (DOCX) [file pone.0242281.s004.docx]

**S2 Table: Reasons for never tested for HIV before and intention to have HIV test in the next 12 month among never testers (by weighted approach)**

|  | 2014  weighted  n=720, 24%  N (%) | 2015  weighted  n=387, 12%  N (%) | 2016  Weighted  n=424, 8%  N (%) | 2017  weighted  n=309, 7%  N (%) |
| --- | --- | --- | --- | --- |
| Reasons for never tested |  |  |  |  |
| Risk Perceptions | 240 (33) | 61 (16) | 85 (20) | 74 (24) |
| Not at risk for HIV (1) | 240 | 50 | 65 | 61 |
| Too young, need consent (11) | 4 | 14 | 20 | 13 |
|  |  |  |  |  |
| Fear | 76 (10) | 34 (9) | 81 (12) | 36 (12) |
| Afraid to learn HIV positive (2) | 70 | 29 | 72 | 27 |
| Afraid of blood, needle or pain (18, 24) | 9 | 5 | 10 | 9 |
|  |  |  |  |  |
| Indifference | 141 (20) | 82 (21) | 76 (18) | 48 (16) |
| Do not want/care (12, 22) | 35 | 25 | 33 | 7 |
| Do not know/think (16, 19) | 107 | 57 | 43 | 41 |
|  |  |  |  |  |
| Discrimination (26) | 26 (3) | 7 (2) | 10 (2) | 9 (3) |
| If HIV+, will lose partner/family friends (3) | 3 | 0 | 1 | 1 |
| If HIV+, will be beaten/hurt by partner (4) | 4 | 3 | 2 | 4 |
| Partner does not want me to test (5) | 11 | 3 | 6 | 3 |
| Family/friends do not want me to test (6) | 8 | 2 | 1 | 1 |
| Wait for partner to test together (17) | 0 | 0 | 0 | 0 |
|  |  |  |  |  |
| Access/Time | 191 (26) | 140 (36) | 92 (22) | 78 (25) |
| Live too far from testing site (7) | 32 | 7 | 6 | 2 |
| Cost too much money to test (8) | 5 | 0 | 0 | 1 |
| Did not know where to test for HIV (10) | 47 | 33 | 38 | 30 |
| Lack of time (13) | 56 | 52 | 21 | 25 |
| Lack of access, opportunity (14, 21) | 48 | 50 | 28 | 19 |
| Lack of knowledge/information (23) | 10 | 0 | 2 | 0 |
|  |  |  |  |  |
| Support | 83 (12) | 37 (9) | 76 (18) | 70 (23) |
| Health provider never offered test (9) | 79 | 37 | 76 | 70 |
| Need encouragement (25) | 9 | 0 | 0 | 0 |
|  |  |  |  |  |
| Intend to test for HIV in the next 12 months |  |  |  |  |
| Yes | 632 (88) | 337 (88) | 339 (83) | 260 (85) |
| No | 79 (11) | 44 (12) | 70 (17) | 46 (15) |

There were 15 never tester participated in more than 1 years.

Participants were allowed to choose more than one reason.
