# Supplementary material for: Using repeated home-based HIV testing services to reach and diagnose HIV infection among persons who have never tested for HIV, Chókwè health demographic surveillance system, Chókwè district, Mozambique, 2014–2017
Source: PLoS One. 2020 Nov 20;15(11):e0242281. doi: 10.1371/journal.pone.0242281 (PMC7678994; doi:10.1371/journal.pone.0242281)
Supplement: S3 Table — (DOCX) [file pone.0242281.s005.docx]

**S3 Table: HBHTC acceptance and HBHTC positive among persons who have not tested before (by weighted approach)**

|  | Never had HIV test before  weighted  N=1826 | Accept HBHTS  weighted  N= 1578 (86 %) | HIV positive by HBHTS  weighted  N=150 (9 %) |
| --- | --- | --- | --- |
| Time |  |  |  |
| 2014 | 720 | 641 (89) | 82 (13) |
| 2015 | 383 | 332 (87) | 36 (11) |
| 2016 | 418 | 344 (82) | 25 (7) |
| 2017 | 305 | 261 (86) | 6 (2) |
| Sex |  |  |  |
| Male | 992 | 839 (85) | 76 (9) |
| Female | 834 | 740 (89) | 73 (10) |
| Age |  |  |  |
| <18 | 826 | 754 (91) | 10 (1) |
| 18-24 | 344 | 312 (91) | 26 (8) |
| ≥ 25 | 655 | 512 (78) | 113 (22) |
| Ever have sex |  |  |  |
| Yes | 1187 | 1005 (85) | 141 (14) |
| No | 632 | 568 (90) | 8 (1) |
| Never asked partner about HIV status when have sex |  |  |  |
| Yes | 770 | 659 (86) | 90 (14) |
| No | 962 | 836 (90) | 47 (6) |
| Reasons for not test |  |  |  |
| Not at risk |  |  |  |
| Yes | 456 | 397 (87) | 24 (6) |
| No | 1370 | 1181 (86) | 125 (11) |
| Fear |  |  |  |
| Yes | 223 | 146 (65) | 18 (8) |
| No | 1604 | 1432 (89) | 132 (9) |
| Indifference |  |  |  |
| Yes | 344 | 283 (82) | 22 (8) |
| No | 1482 | 1295 (87) | 127 (10) |
| discrimination |  |  |  |
| Yes | 52 | 45 (87) | 4 (9) |
| No | 1774 | 1533 (86) | 145 (9) |
| Lack of access/Time |  |  |  |
| Yes | 503 | 473 (94) | 57 (12) |
| No | 1323 | 1105 (84) | 93 (8) |
| Lack of Support |  |  |  |
| Yes | 266 | 241 (91) | 29 (12) |
| No | 1560 | 1337 (86) | 121 (9) |

There were 14 never testers who participated in HPS for 2 year and 1 who participated for 3 yrs. Only the first observation is included in this analysis.
