# Supplementary material for: Using repeated home-based HIV testing services to reach and diagnose HIV infection among persons who have never tested for HIV, Chókwè health demographic surveillance system, Chókwè district, Mozambique, 2014–2017
Source: PLoS One. 2020 Nov 20;15(11):e0242281. doi: 10.1371/journal.pone.0242281 (PMC7678994; doi:10.1371/journal.pone.0242281)
Supplement: Attachment — Submitted filename: Response to Reviewers.docx [file pone.0242281.s011.docx]

**Response to reviewer and academic editor’s comments:**

**Reviewer #1: It is a well written manuscript. The language is simple and easy to understand for readers. However, i believe that the manuscript could be technically more sound especially the methods section. Some steps in the methods section could be explained more vividly to give a better picture of how the study was conducted. Overall, the paper was a good attempt at explaining the HBHTS process**.

We appreciate the favorable comments. We have added a number of clarifications to the Methods section to address other reviewers’ comments, and hope this is sufficient.

**Reviewer #2: The authors report a study titled “Using repeated home-based HIV testing services to reach and diagnose HIV infection among persons who have never tested for HIV, Chókwè Health Demographic Surveillance System, Chókwè District, Mozambique, 2014–2017”, a serial cross-sectional survey.**

**The authors cite a high background HIV prevalence in this setting and yet a large proportion ~ 40% of the PLWHIV have never accessed HIV testing and are therefore unaware that they are HIV-infected and not on readily available life-prolonging ART. This study sought to better understand how to bridge the access/utilization gap through a repeat home-based HIV-testing strategy.**

**With the benefit of survey data from four annual cycles, the research team was able to demonstrate that the highest risk categories of ‘never-tested’ in this study population were adolescents (both boys and girls) and adult men, relative to adult women. Reassuringly, the proportion of never-tested individuals decreased over-time in this relatively stable population; the majority ~ 90% accepted HIV testing and the HIV-positive yield decreased with time. The team concludes that the repeat home -based HIV-testing strategy improved HIV testing in Chókwè study area and recommend implementing similar HIV-testing strategies in high HIV-prevalence settings with low testing coverage, to increase HIV testing uptake and diagnosis among never-tested individuals.**

**This study presents crucial data to better understand effective HIV testing strategies to increase uptake, especially among those persons who have never been tested. In addition to the information value, the manuscript is well written, and therefore should be considered for publication. There were minor observations for consideration/clarification.**

**Methods:**

**Lines 111-114 need revision for grammatical typos.**

These sentences have been revised as follows:

“For participants aged 15-17, parental informed written consent was obtained. For all eligible participants with informed written consent to both the HPS and HBHTS, a 20-30 minute HPS questionnaire was administered by trained interviewers in Portuguese or Shangana before HBHTS was conducted. The study protocol was approved by the CHDSS community advisory board, National AIDS Control Program of the Mozambique Ministry of Health and Mozambique National Committee of Bioethics as research and was determined to be non-engaged research by the CDC Center for Global Health.”

**Statistical analysis:**

**Lines 143-145. “The observed data were analyzed using SAS® (version 9.3, SAS Institute, Inc., Cary, North Carolina, USA) survey procedures that account for correlations among participants within a household following by a sensitivity analysis.”**

**a. It is prudent to indicate the survey method(s) used to account for correlations with citation where applicable.**

**b. Sentence needs revision re: grammar.**

This sentence has been revised, and a citation added, as follows:

“The observed data were analyzed using SAS® survey procedures (PROC SUVEYFREQ and SURVEYLOGISTIC; version 9.3, SAS Institute, Inc., Cary, North Carolina, USA) that account for correlations among participants within a household [26].”

**Lines 147-151. Please indicate the statistical method(s) used for the two sample comparisons of the proportions.**

The lines cited deal only with the descriptive counts and proportions of participants who had never been tested. Comparisons of these proportions were made using logistic regression as described in the next paragraph.

**Results:**

**Lines 203-211. It would be interesting to know how many of those who self-reported never having had sex and accepted testing were HIV-positive?**

Among 585 (all age) participants who self-reported never having had sex and accepted HIV testing, 10 (1.7%) tested positive. Among 535 (aged<18) participants who self-reported never having had sex and accepted HIV testing, 6 (1.1%) tested positive.

**Lines 212-213 need revision. Not clear.**

This sentence has been revised as follows:

“When the data for all 4 survey rounds were combined, HBHTS was accepted by >80% of NTs in each sex and age group, and among NTs reporting each reason for never testing previously, except for fear of needles or testing HIV-positive (Table 4).”

**Discussion:**

**1. Lines 242-249. The authors state in their discussion “These findings suggest that when HBHTS is repeated, screening for sexual risk behavior should be considered to avoid unnecessary costs associated with testing persons who are pre-sexual debut and are likely not to have not been exposed to HIV.”**

**This was overstated and not entirely supported by the data as presented. While, the authors write, “Among NTs aged ≥25 years (97% of whom had sex), the positive yield was ~20% …”, there was no mention of the HIV-positive yield among the 3% self-reported virgins? What about the adolescents who self-reported never had sex?**

**Additionally, self-reported sexual behavior variable has limitations (desirability bias), prone to misclassification of the intended construct. Caution should be exercised in making strong recommendations. Also, please note that there have been occurrences of young adolescents with no sexual experience who were unaware of their perinatally acquired HIV-infection. Lastly, the authors articulate in another section of the discussion about the potential behavior change benefits after a negative test, so it would be counterproductive to deny an HIV-screen test in this high-risk age group under the pretext of a self-reported sexual virginity.**

We appreciate the point you make and have revised this sentence as follows:

“Considering that <2% of participants who self-reported never having had sex and accepted HIV testing tested positive, these findings suggest that when HBHTS is repeated in limited-resource settings, screening for having ever had sex might increase HIV-positive yield and reduce the cost associated with testing persons who are pre-sexual debut and are likely not to have not been exposed to HIV.”

**Academic Editor:**

**Line 43: Can you begin the introduction with a different sentence and then followed by the sentence-"mathematical models and observational studies conclude...."**

We have added the following two sentences to the beginning of the Introduction section.

“HIV remains one of the world’s most serious public health challenges. Approximately, 38 million people worldwide are living with HIV/AIDS and 1.7 million people became newly infected with HIV in 2019 [1].”

**Line 145: Cite a reference for sensitivity analysis.**

The mention of sensitivity analysis has been removed from (what is now) line 148 and a citation added at line 171.

**Line 156: Explain in 1-2 lines how the backward elimination procedure was used to select the final model.**

These sentences have been revised as follows:

“Factors with a P-value<0.1 in the bivariate analyses were included in the initial multivariate models. Backward elimination was used to remove, one at a time, the factor with the highest P-value in the multivariate model until only factors with a P-value<0.05 remained. Two-way interaction terms between the remaining factors were then evaluated and the final model included all terms with a P-value less than 0.05.”

**Line 199: Indifference to what..? State clearly**

We clarified the meaning of indifference by inserting “(i.e., do not know, want to know, care, or think about whether or not they are infected)” in lines 229–230.

**Line 220: Can you elaborate a little on the result of the sensitivity analysis**

The original sentence has been revised and elaborated upon as follows:

“The weighted and unweighted estimates were similar. In particular, the estimated proportions of participants never having been tested by different characteristics and the proportions of HBHTC acceptance and HBHTC positive among persons who have not been tested obtained using the two approaches were very close. In addition, the factors associated with never tested were the same with the two approaches.”
