# Supplementary material for: Using repeated home-based HIV testing services to reach and diagnose HIV infection among persons who have never tested for HIV, Chókwè health demographic surveillance system, Chókwè district, Mozambique, 2014–2017
Source: PLoS One. 2020 Nov 20;15(11):e0242281. doi: 10.1371/journal.pone.0242281 (PMC7678994; doi:10.1371/journal.pone.0242281)
Supplement: Attachment — Submitted filename: Response to Reviewers.docx [file pone.0242281.s012.docx]

**Response to the editor:** Thank you very much for your comments. Please see the responses below.

1. **Repeat HBHTS is a useful and powerful approach to reach non-testers. However, home-based HIV testing comes at a cost and most programs, including governments -- especially in LMICs -- might not be able to implement HBHTS without additional donor support. I suggest that the authors include a discussion around the cost of HBHTS vis-a-vis the scalability and sustainability of such programs in poor-resource settings.**

Thanks for the recommendation. We have added the following paragraph to the Discussion Section.

“Affordability, feasibility, and sustainability are key for the success of implementing health programs. Costs-per-person tested and counselled and costs-per-person tested HIV positive are important cost measures for policy makers to decide if the programs can reach the objectives of testing (i.e., increasing HIV testing coverage, identifying PLHIV who were not aware of their status and linking the newly diagnosed PLHIV to treatment). Hauck (2019) reviewed the cost of HBHTS studies in sub-Saharan Africa and concluded that the average cost per person tested for HBHTS was $23 (range: $6 to $55); the average cost per person tested HIV-positive was $439 (range: $66 to $800) [39]. HBHTS was found to be less costly and more effective than FBHTS in increasing HIV testing uptake in rural South Africa [40]. Identifying an optimal frequency of HBHTS has also been discussed. HIV positive yield in the first round depends on the level of prevalence and the testing coverage by existing HIV testing modalities (e.g., FBHTS). The yield of subsequent rounds depends on the HIV incidence and the testing uptake of the prior rounds and other existing modalities. The costs-per-person HIV positive would increase unless HIV uptake among PLHIV were higher in the subsequent rounds [39]. Modeling studies has been done to estimate the optimal frequency of HBHTS [41-43]. Diminishing returns have been suggested but none of the studies offer firm recommendations [39].”

2. **The authors report that the HIV yield reduced from 13% in 2014 to 2% in 2015-17. Similar reductions were observed across sex and age-groups. I expected to see a clear discussion on why HIV prevalence declined significantly in the four-year period. Definitely, I would imagine that HBHTS is not the reason for the decline; so, what caused the decline? Even if the authors did not set out to explore why the HIV yield declined, it would be important to discuss what could have caused the observed decline in the HIV yield over time. Is it that HBHTS reached less risky individuals over subsequent rounds? Characteristically speaking, would the authors argue that NTs were generally a high-risk group (initially, e.g. in 2014) but that the demographic profile changed with continued repeat HBHTS?**

The homebased HIV testing program was part of HIV combination prevention programs and other HTS delivery strategies such as routine HIV testing at district health facilities; school-based and outreach testing were also being implemented. The population profile that HBHTS reached was similar overtime.

However, the population profile of never testers did change over time: there were more younger people (age<18) and more participants who reported never having had sex. On the other hand, the estimated prevalence overall in Chokwe ranged from 28% to 25% during the study period. So, yes, HBHTS reached less risky individuals over subsequent rounds. As we discussed in the Discussion Section:

“HIV-positive yield among NTs decreased from 13% during 2014 to 2% during 2017. The decrease in positive yield coincided with a decreasing proportion of NTs who had ever had sex (from 80% during 2014 to 65%, 57%, and 33% in subsequent years) and an increasing proportion of NTs who were aged <18 (from 29% during 2014 to 46%, 58%, and 79% in subsequent years). Among NTs aged ≥25 years (97% of whom had sex), the positive yield was ~20% during the first 3 years, decreasing to 6% during 2017. Considering that <2% of participants who self-reported never having had sex and accepted HIV testing tested positive, these findings suggest that when HBHTS is repeated in limited-resource settings, screening for having ever had sex might increase HIV-positive yield and reduce the cost associated with testing persons who are pre-sexual debut and are likely not to have not been exposed to HIV.”

3. **Related to item 2 above; as the HIV yield reduces significantly, the cost per HIV-positive person identified increases subsequently. I think this aspect should be discussed along with the authors' response to item 1 above.**

This is a good point. Please see our addition to the discussion in our response to item 1.

4. **In line 243, the authors should use 'were' in place of 'was' after '...high prevalence settings...'**

Corrected. Thank you!

5. **In lines 256-57, reference is made to 6% during 2017 but in lines 218-19, this percentage is given as 5%. The authors should harmonize these figures.**

Corrected. Thank you!
