# Supplementary material for: Using repeated home-based HIV testing services to reach and diagnose HIV infection among persons who have never tested for HIV, Chókwè health demographic surveillance system, Chókwè district, Mozambique, 2014–2017
Source: PLoS One. 2020 Nov 20;15(11):e0242281. doi: 10.1371/journal.pone.0242281 (PMC7678994; doi:10.1371/journal.pone.0242281)
Supplement: Attachment — Submitted filename: Response to Reviewers.docx [file pone.0242281.s013.docx]

**Response to the editor:** Thank you very much for the comments. Please see below.

1. **PLoS ONE uses the Vancouver style while formatting references. The authors should check refs 9,27, 39 and 43 and ensure these are well aligned to the journal's referencing style. The authors may find it helpful to visit this link for additional information:** [**https://journals.plos.org/plosone/s/submission-guidelines#loc-references**](https://journals.plos.org/plosone/s/submission-guidelines#loc-references)

The references are checked and updated to be aligned to the journal’s referencing style. Please see the updated manuscript.

2. **In ref 40, the journal name is not provided.**

The journal name is added. Thank you!!

3. **The authors should ensure that journal names are written in a consistent way, for example, in ref 4, the journal name is given as "PLoS One"; while in ref 8, the same journal is given as "Plos one". The authors should check other journal names to correct them accordingly.**

The journal names are checked and updated. Thank you!!
